# A Case Report of Vitamin C Deficiency Mimicking Osteomyelitis

**DOI:** 10.3390/pediatric18020057

**Published:** 2026-04-14

**Authors:** Akash Daswaney, Nirali Borad, Anhthu Trinh, Stephanie Thompson, Youmna Mousattat

**Affiliations:** 1 Pediatrics, Women and Children’s Hospital, Charleston Area Medical Center (CAMC), Charleston, WV 25302, USA; atrinh@usf.edu (A.T.); ymousattat@hsc.wvu.edu (Y.M.); 2 Pediatrics, West Virginia University-Charleston Division, Charleston, WV 25304, USA; 3 Pediatric Pulmonology, Nationwide Children’s Hospital, The Ohio State University College of Medicine, Columbus, OH 43205, USA; 4 Pediatrics, West Virginia University, WVU Medicine Golisano Children’s Hospital, Morgantown, WV 26505, USA; 5 Medicine-Pediatrics, University of South Florida, Tampa, FL 33606, USA; 6 CAMC Institute for Academic Medicine, Charleston Area Medical Center, Charleston, WV 25304, USA; stephanie.thompson2@vandaliahealth.org

**Keywords:** vitamin C deficiency, osteomyelitis, MRI, developmental delay

## Abstract

Vitamin C, also known as ascorbic acid, plays a pivotal role in forming blood vessels, cartilage, muscles, and collagen in bones. We report a 6-year-old non-verbal female with global developmental delay who presented with complaints of lower limb pain and inability to bear weight. Symptoms started five weeks prior to presentation and had progressed from decreased activity to complete loss of weight-bearing. Physical examination showed gingival hyperplasia, perifollicular petechiae, lower limb edema, and corkscrew hair. Initial radiologic findings raised concerns of osteomyelitis, showing bone marrow edema, periosteal reaction, and cortical irregularity. However, correlation with dietary history limited to flavored milk and yogurt and lacking fruits and vegetables, in conjunction with clinical presentation, suggested vitamin C deficiency, and she was started on ascorbic acid. Vitamin C deficiency was later confirmed on day 7 by a low C deficiency level (<0.1 mg/dL). Treatment with ascorbic acid, multivitamins, and supportive therapy led to gradual recovery, and gastrostomy tube placement facilitated supplementation. This case highlights the importance of detailed dietary history and recognition of clinical signs of vitamin C deficiency. Early dietary assessment and clinical correlation can prevent unnecessary invasive procedures and prolonged antibiotic therapy. Early identification enables timely intervention, reducing morbidity and improving quality of life.

## 1. Introduction

Vitamin C deficiency is a nutritional insufficiency caused by inadequate vitamin C intake that can result in serious musculoskeletal, dermatologic, and hematologic complications. The condition has been described since ancient times, including in Egypt around 1500 BC, and was formally characterized in 1747 by Sir James Lind, who showed that citrus fruits could prevent and treat vitamin C deficiency [1,2].

Although once considered rare in high-income countries, recent reports show a rising incidence of vitamin C deficiency, particularly among children with atypical or restrictive diets [1,2]. Children with neurodevelopmental disorders, including autism spectrum disorder and global developmental delay, are especially vulnerable due to highly selective eating patterns and difficulties communicating symptoms. Other risk factors include psychiatric eating disorders, food insecurity, neglect, dependence on enteral feeds, gastrointestinal malabsorption, end-stage renal disease, and iron overload from repeated transfusions [3,4]. Even when children consume adequate calories, their diets can often lack essential micronutrients. Vitamin C stores can be depleted within one to three months without sufficient intake.

This case is distinguished by the degree to which vitamin C deficiency radiologically and clinically mimicked osteomyelitis, resulting in diagnostic delay across multiple care settings and prompting consideration of invasive and antimicrobial therapies. A detailed dietary history, often overlooked in outpatient and inpatient evaluations, provided the initial clue to nutritional deficiencies. Thus, clinicians should remain vigilant for vitamin C deficiency as this potentially fatal but easily treatable condition can be overlooked due to nonspecific symptoms, subsequently leading to delayed diagnosis, unnecessary investigations, and preventable complications.

## 2. Case Presentation

A 6-year-old female with a past medical history of septo-optic dysplasia, autism spectrum disorder, growth hormone deficiency, and global developmental delay was admitted to the pediatric floor for further diagnostic workup to identify the cause of her loss of ambulation. In the month preceding admission, the patient developed decreased activity and sociability, ultimately progressing to complete cessation of walking, with her parents carrying her for all activities of daily living. Additionally, during this time, she underwent multiple outpatient evaluations, including the emergency department (ED) and an orthopedic clinic. Five weeks prior to inpatient admission, she presented to the ED with decreased mobility, inability to bear weight on her lower limbs, oral cavity pain, foul-smelling urine, and constipation. A urinary tract infection (UTI) was suspected, and laboratory workup, including a complete blood count (CBC) and comprehensive metabolic panel (CMP), was unremarkable except for an elevated C-reactive protein (CRP) of 6 mg/dL. X-rays of the bilateral hips and legs were normal. Examination revealed a palpable stool burden without oral lesions. She was discharged with cephalexin, empirically for a presumed UTI, and polyethylene glycol, with follow-up advised with her primary care physician (PCP) and dentist.

On follow-up with her PCP two days post-ED visit, she continued to have persistent symptoms. Diffuse soft tissue swelling of both lower limbs was noted, and she was referred to an orthopedic clinic, where a lower extremity cast was applied. Over the next week, her mother observed worsening swelling. After the cast was removed, significant edema with pinpoint petechiae was noted. During the same period, she underwent dental care, including a tooth extraction. She experienced a brief self-limited fever and persistent petechiae, but coagulation studies remained normal.

At the end of this month-long period, the child had an appointment at a pediatric neurology clinic due to the continued inability to walk. The pediatric neurology clinic was unable to identify the cause of her inability to ambulate and advised admission to the pediatric floor for further evaluation. On inpatient admission, she was distressed but hemodynamically stable. A detailed dietary history revealed exclusive intake of chocolate milk and yogurt, with cessation of pureed fruits and vegetables several months earlier. Physical examination showed corkscrew hair, gingival hyperplasia, a non-blanching petechial rash extending from the ankles to mid-calf, bilateral lower extremity pitting edema, pain with passive movement, inability to bear weight, baseline horizontal nystagmus, and a palpable fecal burden in the left lower quadrant. Initial labs reflected a chronically malnourished state; microcytic hypochromic anemia, hypoalbuminemia, and elevated CRP (Table 1).

**Figure 1:**
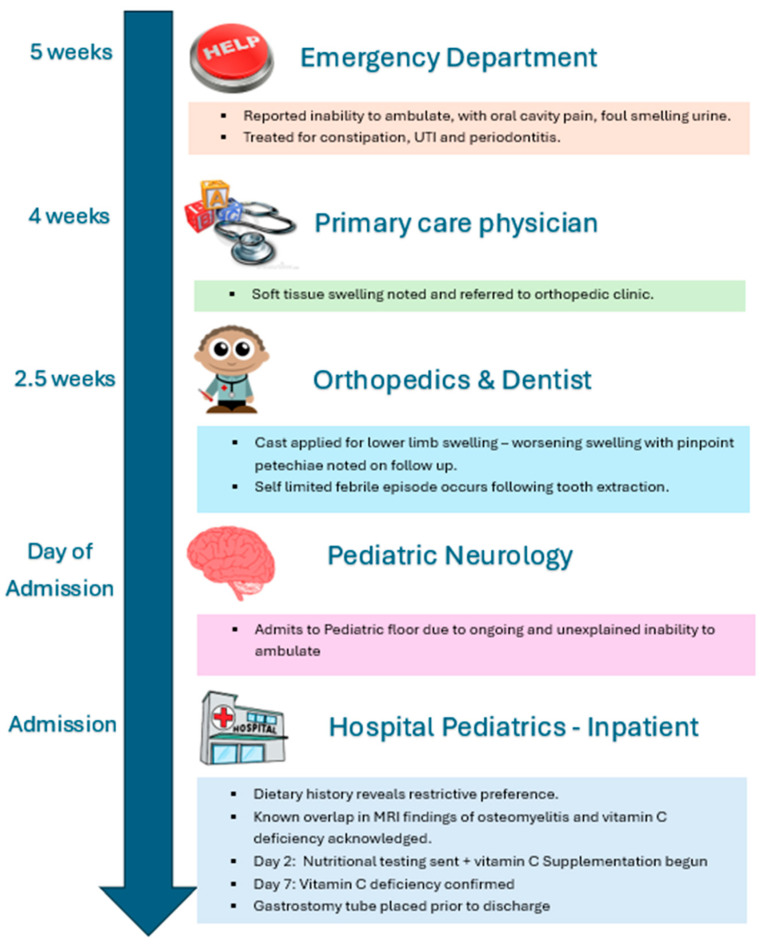
Clinical timeline and diagnostic course.

**Figure 2:**
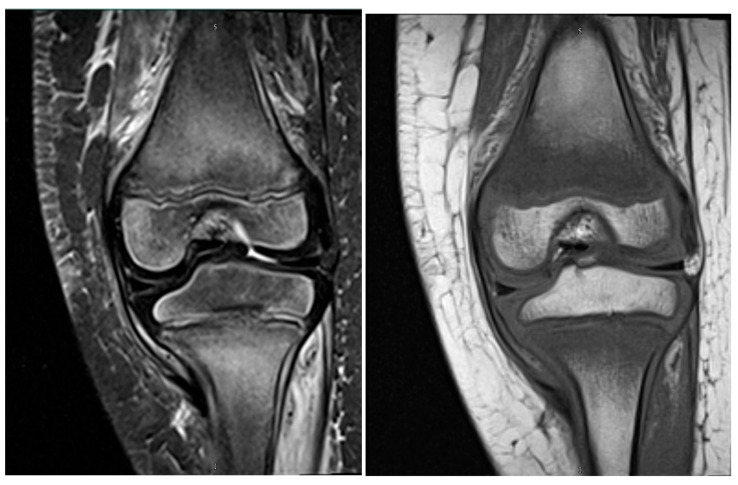
MRI of lower extremities shows abnormal STIR signals with metaphyseal/physeal involvement, periosteal reactions, and small joint effusions (**left**). Hypointense T1-weighted images depicting the same are present (**right**).

Blood product transfusion was deferred due to the absence of signs suggestive of symptomatic anemia (hemodynamic instability, chest pain, altered mental status, headaches). Given the clinical presentation (Figure 1), non-accidental trauma, infection (including osteomyelitis), malignancy, and nutritional deficiencies were considered in the differential diagnosis. Radiological investigations were done early in the evaluation. Repeat X-rays of the knees, hips, and ankles remained normal, and abdominal and lower extremity ultrasounds excluded deep vein thrombosis, soft tissue masses, or neuroblastoma. Magnetic resonance imaging (MRI) revealed findings highly suggestive of osteomyelitis: abnormal short tau inversion recovery (STIR) signal, hypointense T1-weighted images, and enhancement in the proximal metaphysis and physis of the femur, tibia, and fibula bilaterally, with periosteal reactions and joint effusions. (Figure 2; Table 2).

However, osteomyelitis diagnosis did not explain our patient’s entire clinical picture. Furthermore, our patient’s dietary history and physical examination features pointed towards nutritional deficiency. This corroborated laboratory findings (elevated ESR and CRP with a normal procalcitonin level) that were suggestive of an inflammatory rather than an infectious etiology. We decided not to administer antibiotics and instead re-engaged in a discussion with the radiology team. The care team discussed the patient’s constellation of symptoms; the symmetric pattern of marrow involvement on imaging, absence of systemic signs of infection, and markedly restricted diet. A known radiologic overlap between vitamin C deficiency and osteomyelitis was acknowledged, strengthening the belief that vitamin C deficiency was responsible for the patient’s symptoms of self-limiting bleeding episodes, lower extremity swelling, and loss of ambulation. Chronic dairy consumption likely contributed to our patient’s anemia and caused constipation. Based on this diagnostic thought process, blood investigations for nutritional deficiencies were promptly obtained on day 2 of admission. We initiated a treatment regimen consisting of intravenous fluids, analgesics, ferrous sulfate, and ascorbic acid 250 mg twice daily (BID), administered orally or via nasogastric tube. Physical therapy was initiated, and nutritional support was provided. A formal swallowing assessment, speech language pathology evaluation or video-fluoroscopic swallow study was deferred as the patient’s nutritional deficiency was related to restricted dietary interest rather than aspiration or swallowing dysfunction. With the help of a dietitian, a diet consisting of fortified chocolate-flavored protein shakes was instituted to accommodate our patient’s restricted interest.

On day seven of admission, vitamin panel results confirmed severe vitamin C deficiency (<0.1 mg/dL; normal >0.6 mg/dL), along with folic acid and iron deficiencies. By this time, the patient had already begun to show gradual but consistent improvement in ambulation, and her rash was subsiding. At the time of discharge, she continued to express severe dietary selectivity. In view of this, and the need for reliable nutritional supplementation and medication administration, a gastrostomy tube was placed prior to discharge. The patient tolerated the procedure well without complications. Upon discharge, she was prescribed ascorbic acid 250 mg BID for 90 days, multivitamins, ferrous sulfate, folic acid, and famotidine. At follow-up visits, she demonstrated steady recovery, and two months post-admission, she was asymptomatic and had returned to baseline ambulation, highlighting the rapid clinical response to vitamin C supplementation.

## 3. Discussion

Centuries ago, a mutation impairing the conversion of glucose to ascorbic acid made vitamin C an essential exogenous vitamin [1]. Vitamin C deficiency, once associated with 17th-century sailors, and later with 19th-century urban populations reliant on proprietary foods [3], has reemerged in modern times, particularly among children with restrictive diets, sensory sensitivities, or neurodevelopmental disorders. These children may face feeding challenges, limited dietary variety, and difficulty communicating symptoms, all of which contribute to increased vitamin C deficiency risk [5,6]. Vitamin C is a water-soluble electron donor that plays a key role as an antioxidant and in collagen synthesis. Its deficiency can cause ossification failure and capillary endothelial abnormalities that are present in distinct clinical stages, including oral lesions, cutaneous manifestations, musculoskeletal disease, and generalized asthenia [1,2,4]. Symptoms usually become clinically relevant after one to six months of deficiency and may include gingival bleeding, sub-follicular/periosteal hemorrhages, ‘corkscrew’ hair, anemia, petechiae, poor wound healing, dry skin, oral ulcers, and vascular purpurae [2,3,7].

Maintaining adequate vitamin C levels can be particularly challenging in children with autism spectrum disorder, given their restricted diets, selective food preferences, and frequent musculoskeletal complaints [5,6,8]. Defective collagen synthesis in these children predisposes them to avulsion of the periosteum, which is loosely attached, inhibiting proper skeletal tissue growth [9,10]. Therefore, the prevalence of vitamin C deficiency is higher in children with restrictive diets, food allergies, eating disorders, or other developmental delays compared to the general US pediatric population [11]. The vague presentation of vitamin C deficiency symptoms, such as myalgia, arthralgia, and lower limb weakness, along with multisystem involvement, makes diagnosis challenging. When a pediatrician encounters inability to ambulate or bear weight, emergent conditions such as osteomyelitis, spinal cord compression, septic arthritis, and malignancy should be considered [12]. Other differentials include non-accidental trauma, fractures, Guillain-Barre syndrome, transverse myelitis, growing pains, joint hypermobility, psychogenic disorders, and inflammatory diseases [13,14]. A thorough clinical assessment remains critical in narrowing the diagnosis, and vitamin C, though rarely monitored compared to some other micronutrients, can be easily measured when deficiency or insufficiency is suspected. Severe vitamin C deficiency (scurvy) levels are below 0.2 mg/dL, while hypovitaminosis C is characterized by 0.2–0.6 mg/dL. Levels of 0.9 mg/dL are considered ‘adequate’, whereas optimal or tissue saturation levels are reached with levels exceeding 1.0 mg/dL [15]. Recent pharmacological reviews emphasize that vitamin C plays diverse metabolic roles beyond its classical antioxidant function, and clinically significant deficiency can occur across a spectrum of these levels [16]. This highlights the importance of awareness and maintenance of adequate vitamin C levels, particularly in high-risk pediatric populations.

In our case, nutritional deficiency was considered, but the vitamin C deficiency diagnosis was initially overlooked because radiologic and laboratory investigations were prioritized over a comprehensive dietary assessment. The radiologic overlap between vitamin C deficiency and osteoarticular infection can contribute to diagnostic uncertainty and delayed recognition [9,17]. While classic X-ray findings of vitamin C deficiency, such as diffuse osteopenia, subperiosteal hemorrhages, and metaphyseal changes including Frankel’s line, Trummerfeld zone, Pelkan spurs, and Wimberger’s ring, were absent, MRI showed symmetric marrow edema, abnormal STIR signal, metaphyseal involvement, subperiosteal hemorrhage, and soft tissue changes, mimicking osteomyelitis. However, the bilateral symmetric distribution of metaphyseal marrow edema with subperiosteal hemorrhage is more characteristic of vitamin C deficiency and helps distinguish it from infectious osteomyelitis, which typically presents with unilateral, focal marrow involvement and systemic inflammatory findings. Recognition of nutritional risk factors in the patient guided the diagnosis and prevented unnecessary interventions. Suspicion of vitamin C deficiency and subsequent treatment prevented unnecessary interventions such as a prolonged course of broad-spectrum antibiotics, further imaging and a possible bone biopsy. From this, one can even infer that an awareness of classical vitamin C deficiency X-ray findings can also facilitate prompt diagnoses when first-line investigations are obtained [8,9,10].

Another important differential is chronic recurrent multifocal osteomyelitis (CRMO), which can resemble vitamin C deficiency both clinically and radiologically. It typically presents with multifocal inflammatory bone lesions on T2/STIR sequences, often following a relapsing–remitting course and shows minimal soft tissue involvement (no hemorrhagic periosteal collections) [18,19]. In our patient, the lack of systemic infection signs, together with clear nutritional risk factors, made vitamin C deficiency more likely than either infectious osteomyelitis or CRMO.

This case underscores the importance of considering nutritional deficiencies in children with chronic medical conditions, restrictive diets, or underlying malignancies. Although vitamin C deficiency presenting with musculoskeletal findings that can mimic osteomyelitis has been described in the literature, this case highlights how the diagnosis can still be overlooked in clinical practice. Ultimately, vitamin C deficiency remains primarily a clinical diagnosis, guided by careful clinical assessment and recognition of characteristic clinical and radiologic features. Additionally, our case demonstrates how a diagnosis can be missed not because it is rare or complex, but because a basic clinical step was overlooked. The patient was evaluated multiple times across different care settings, yet the underlying cause remained unrecognized, highlighting a critical blind spot in routine practice. Patterns such as extreme food selectivity, long-term avoidance of fruits and vegetables, or reliance on processed foods often provide the earliest and most dependable clues to nutritional deficiency. When these clues are missed, nutritional conditions can mimic infections, rheumatologic disease, or even malignancy, prompting unnecessary and invasive diagnostic evaluations, leading to unnecessary treatments. In our patient, this led to avoidable lower limb casting and repeated specialty referrals. Thus, although the clinical presentation of vitamin C deficiency has been previously described, this case highlights a continuing diagnostic vulnerability in everyday clinical care. If left undiagnosed, nutritional deficiencies can lead to serious and potentially life-threatening complications, including infections, bleeding, physical disability, and even death [20]. Pediatricians should recognize high-risk children and provide guidance on diet, supplementation, and overall care. Routine dietary assessment is essential to improve diagnosis, prevent harm, and reduce unnecessary healthcare use.

## 4. Conclusions

Vitamin C deficiency should be considered in any child presenting with unexplained musculoskeletal pain, particularly those with restrictive diets or neurodevelopmental disorders. This case expands upon prior literature by demonstrating that, in addition to dietary history, careful clinical interpretation of MRI findings is essential. Integrating imaging results with the patient’s overall presentation and maintaining a high index of suspicion allows for timely diagnosis and avoidance of unnecessary invasive management. A focused dietary assessment remains a pivotal component of evaluation and can facilitate timely recognition of vitamin C deficiency, even when imaging findings appear alarming. Prompt supplementation leads to rapid clinical improvement and prevents long-term complications, reinforcing the value of history-driven, cost-conscious, and imaging-contextualized pediatric care.

## Figures and Tables

**Table 1:** Laboratory findings show iron deficiency anemia, hypoalbuminemia, and hyponatremia.

Complete Blood Count (CBC)		Reference Values
Hemoglobin (g/dL)	7.2 LOW	11.2–14.7 g/dL
Hematocrit (%)	19.7 LOW	34–43.8%
Mean corpuscular volume (MCV)	75.1 LOW	76–90.1 fL
White blood cells (WBC)	8.1	4.2–13.2 × cells/mm^3^
Platelet count	325	163–461 × 10^9^/L
Red cell distribution width (RDW)	13.8	10.7–14%
Reticulocyte Count	6.3 HIGH	0.5–2.1%
Comprehensive Metabolic Panel (CMP)		
Sodium (Na)	130 LOW	134–142 mmol/L
Potassium (K)	4.3	3.5–4.9 mmol/L
Chloride (Cl)	101	96–111 mmol/L
Bicarbonate (HCO3)	19	18–29 mmol/L
Blood urea nitrogen (BUN)	15	4–18 mg/dL
Creatinine (Cr)	0.3	0.2–0.7 mg/dL
Glucose	139 HIGH	67–138 mg/dL
Calcium (Ca)	8.6	8–10.4 mg/dL
Aspartate transaminase (AST)	25	11–47 U/L
Alanine transaminase (ALT)	20	5–39 U/L
Alkaline phosphatase (ALP)	144	59–291 U/L
Total protein	6.6	4.9–8.3 g/dL
Albumin (g/dL)	2.8 LOW	3.5–5.5 g/dL
Total bilirubin	0.3	0.2–0.9 mg/dL
Inflammatory Markers		
C-reactive protein (CRP)	34.1 HIGH	<5 mg/dL
Erythrocyte sedimentation rate (ESR)	50 HIGH	<13 mm/hr
Procalcitonin	0.26	0.05–0.5 ng/mL
Coagulation profile		
Activated partial thromboplastin time (APTT)	28.7	25.1–36.5 s
Prothrombin time (PT)	13.2 HIGH	9.4–12.5 s
International normalized ratio (INR)	1.17	>3.99
Other		
Fecal Occult Blood Testing (FOBT)	Negative	
Creatine kinase (CK)	37	47–515 U/L
Lyme Disease Antibody	Negative	

**Table 2:** Radiological investigations (X-rays and MRIs of lower extremities and spine) show no fractures but findings suggestive of hypovitaminosis C and initially concerning for osteomyelitis, including cortical thickening, marrow edema, periosteal reactions, and effusions.

Modality	Region	Key Findings
X-ray	Lower extremities (Femur, Knee, Tibia/Fibula, Ankle)	No fracture, dislocation, or radiopaque foreign body
	Hips (Bilateral)	Normal femoral capital epiphyses; no fracture/dislocation; mild cortical thickening of proximal medial left femur (unchanged); no lytic or destructive lesions
MRI	Hips with and without contrast	Edema-like signals in pelvis; bilateral hip effusions; musculature edema (possible myositis/denervation/strain); moderate left and mild right greater trochanteric bursitis (possible infection); diffuse red marrow hyperplasia (likely anemia-related)
	Left Knee with and without contrast	Abnormal STIR bright and low T1 signals in distal femur, proximal tibia/fibula metaphyses/physes; periosteal and subperiosteal changes; small joint effusion; initially concerning for osteomyelitis
	Left Ankle with and without contrast	Mild edema-like signals in the peripheral portions of the bone of the entire foot and ankle, raising concerns for osteomyelitis
	Lumbar Spine with and without contrast	No acute abnormality. No evidence of discitis osteomyelitis
MRI addendum	Lower extremities	Findings compatible with vitamin C deficiency: diffuse marrow edema, periosteal reactions, subperiosteal hemorrhage in proximal femurs, and muscular signal abnormalities; nutrient deficiencies (vitamin C, vitamin D, anemia) likely contributing to osseous insufficiency; non-accidental trauma unlikely; osteomyelitis less likely given history, negative blood cultures, and absence of fever

## Data Availability

The original contributions presented in this study are included in the article. Further inquiries can be directed to the corresponding authors.

## Abbreviations

The following abbreviations are used in this manuscript:

ED	Emergency Department
UTI	Urinary Tract Infection
CBC	Complete Blood Count
CMP	Complete Metabolic Panel
CRP	C-reactive Protein
PCP	Primary Care Physician
MRI	Magnetic resonance imaging
STIR	Short Tau Inversion Recovery
BID	Twice Daily
CRMO	Chronic Recurrent Multifocal Osteomyelitis
